# A review of *APC* somatic mosaicism and specific *APC* variants - I1307K and promotor variants

**DOI:** 10.1007/s10689-025-00464-w

**Published:** 2025-04-16

**Authors:** Shira Shur, Anna K. Sommer, Andrew Latchford, Isabel Spier, Lior H. Katz

**Affiliations:** 1 The Gonczarowski Family Institute of Gastroenterology and Liver Diseases, Shamir Medical Center, Zerifin, Israel; 2 Institute of Human Genetics, Medical Faculty University of Bonn, Bonn, Germany; 3 Polyposis Registry, Centre for Familial Intestinal Cancer, St Mark’s Hospital, London, UK; 4 Department of surgery and cancer, Imperial College London, London, UK; 5 National Center for Hereditary Tumor Syndromes, University Hospital Bonn, Bonn, Germany; 6 Department of Gastroenterology, Hadassah Medical Center, Hadassah Ein-kerem, Jerusalem, Israel; 7 Faculty of Medicine, Hebrew University of Jerusalem, Jerusalem, Israel

**Keywords:** Familial adenomatous polyposis (FAP), *APC*, Mosaicism, I1307K, Colorectal cancer risk, Gastric adenocarcinoma and proximal polyposis of the stomach (GAPPS)

## Abstract

In the majority of patients with a classical Familial Adenomatous Polyposis (FAP) a pathogenic *APC* germline variant is identified; usually these are truncating variants in the coding region of *APC.* However, there are some special circumstances in which FAP is not the result of a pathogenic heterozygous germline variant in *APC* (mosaicism) and tspecific *APC* variants which do not cause FAP (I1307K and promotor variants). This paper will discuss these three conditions. *APC* somatic (postzygotic) mosaicism can be identified in up to 50% of unexplained adenomatous polyposis cases. The ability to identify *APC* postzygotic mosaicism depends on the the detection method (today usually next-generation sequencing) and also the tissue being analysed (investigation of multiple colorectal adenomas is more sensitive than leukocyte DNA). Identifying mosaicism has important implications in terms of an individual’s management and managing risk in family members. The I1307K variant in *APC* is prevalent among Ashkenazi Jews (AJ) but can also be found in Sephardi Jews and individuals of non-Jewish descent. While this variant does not cause polyposis, it increases the risk of colorectal cancer (CRC) by 1.68-fold in AJ individuals. However, the link between the I1307K variant and CRC risk in non-AJ populations, is less well-established. Furthermore, its potential impact on other types of cancer remains unclear. Consequently, the classification of this variant, along with appropriate screening and surveillance recommendations, remains a subject of ongoing debate among leading medical and genetic organizations. Variants in the *APC* promotor 1B region cause the relatively newly described condition of gastric adenocarcinoma and proximal polyposis of the stomach (GAPPS). It is said to have an isolated gastric phenotype, with neither duodenal, large bowel nor extra-intestinal manifestations. There are many uncertainties regarding this condition, it’s penetrance and management. Lack of clinical data and poor understanding of the natural history of the condition remain significant barriers to developing guidelines to manage this condition.

## *APC* MOSAICISM

It is well known that the impact of somatic mosaicism is underestimated in hereditary (tumor) syndromes particularly those with a high *de novo* mutation rate such as Familial Adenomatous Polyposis (FAP) or neurofibromatosis type 1 (NF1) [1–7]. Somatic mosaicism means that only a part of the somatic cells carry the same (likely) pathogenic variant. Since such variants occur usually postzygotically during embryonic development, also the terms postzygotic variants / mosaics are used. Postzygotic mosaics are increasingly being recognised as the cause of genetic diseases, which is due not only to greater clinical awareness but also to more sensitive detection methods. In addition, the identification of a mosaic has significant consequences for the management of the individual with mosaicism and managing the wider family.

*APC* somatic (postzygotic) mosaicism was reported in up to 50% of unexplained adenomatous polyposis cases and is mainly detected in patients with an attenuated polyposis phenotype. A review of *APC* mosaicism was published by Jansen and Goel in 2020 [8] based on a systematic literature search: 57 patients carrying *APC* mosaic variants are described so far in 19 publications, 8 of these are case reports.

**Fig. 1:**
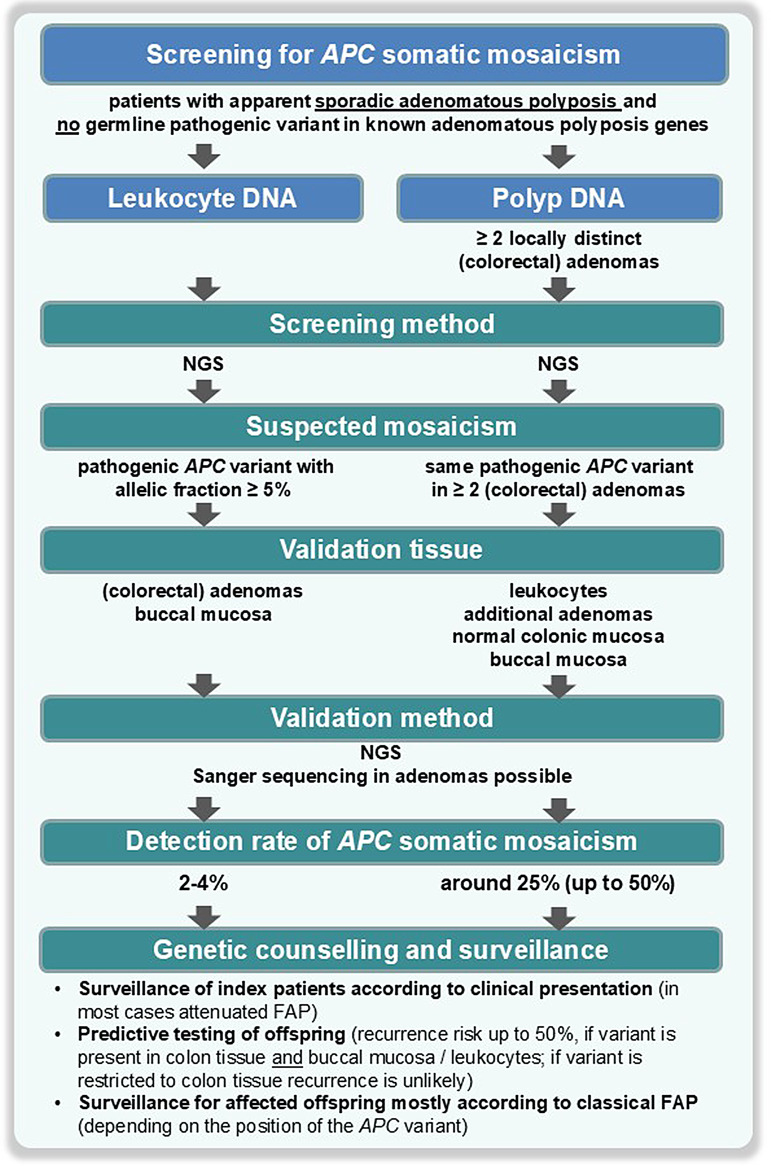
Suggested procedure of screening for APC somatic mosaicism; if no somatic variant can be identified by screening of leukocyte DNAthen a screening of at least two adenomas can be subsequently performed. NGS = next generation sequencing

The identification rate of *APC* mosaicism depends on the patient cohort / phenotype, the diagnostic methods and the investigated tissues, a suggested procedure of screening for *APC* mosaicism based on current technology is shown in Fig. 1. The first two studies evaluating *APC* mosaicism in larger cohorts detected *APC* mosaicism in 10–20% of leukocyte DNA among a selected group of unrelated patients with suspected or confirmed *de novo* pathogenic variants in *APC* [9, 10]. Of index patients with unexplained (no detectable pathogenic variant in *APC* and *MUTYH*) and apparent sporadic disease, the frequency of somatic *APC* mosaicism isestimated to be around 2–4% [11]. Additionally, the selection of diagnostic methods plays an important role. Traditional methods such as the protein truncation test (PPT), denaturing high performance liquid chromatography (DHPLC) and denaturing gradient gel electrophoresis (DGGE) are more sensitive for the detection of mosaics than Sanger sequencing [9, 10]. The detection threshold of Sanger sequencing is about 10–15% mutated alleles. This suggests that a number of mosaic cases are likely to be overlooked in series where only Sanger sequencing has been used [9, 12]. In the era of next-generation sequencing (NGS) constitutional testing may still miss mosaicism; t mosaics confined to colon tissue may not be detectable or easily missed in data from leukocyte-derived DNA. Adding the complexity of interpreting NGS results is, that when pathogenic variants are found at a level below 5% in leukocyte-derived DNA, this is not reliable and may represent a false positive result [11].

A more stringent approach to elucidate *APC* mosaicism in unexplained adenomatous polyposis patients without a family history, is to sequence multiple (≥ 2) samples of affected tissue from the same individual, in this case usually colorectal adenomas, using NGS to search for the same pathogenic *APC* variant (Fig. 1). Since fresh tissue is often not available, usually formalin-fixed paraffin-embedded tissue (FFPE) is used. As the quality of DNA extracted from FFPE material is often limited, the detection of (low-grade) mosaics might be restricted. To avoid clonally related adenomas, these should be anatomically separated (i.e. they should not be directly adjacent or originate from a single laterally spreading lesion). Based on this approach *APC* somatic mosaicism was identified in 25% (5/20) of patients [11]. In 2/5 cases, the mosaic level in leukocyte DNA was slightly below the sensitivity threshold of Sanger sequencing; while in 3/5 cases, the allelic fraction was either very low (0.1-1%) or the pathogenic variants were not detectable. In two cases also the upper gastrointestinal tract was affected. Interestingly, the majority of mosaic pathogenic variants were located outside the somatic *Mutation Cluster Region* (codons 1286–1513) of the *APC* gene [13]. Similar multiple adenoma approaches were also used in the studies by Jansen et al. [14] and Ciavarella et al. [15], in which *APC* mosaicism could be identified in 50% of unexplained (attenuated) adenomatous polyposis patients (9/18 and 4/8, respectively). In the latter study digital PCR (dPCR) was used as another sensitive method to confirm mosaicism in further tissues. In another study high resolution melting (HRM ) was used for screening and *APC* mosaicism could be detected in 2/161 (1%) leukocyte DNA samples and 2/2 (100%) tumor DNA samples [16].

The diagnosis of mosaicism is also important in terms of estimating the risk for first degree relatives (Fig. 1). While siblings and parents will not be affected, the risk for children is up to 50%, depending on the stage of embryonic development at which the variant arose [1, 8]: Primordial germ cells are thought to arise from the ectodermal layer during the second week of embryogenesis. Variants present in the endodermal and ectodermal layers, represented by colon tissue and buccal mucosa, respectively, are presumed to have arisen before day 8 and are therefore more likely to be inherited by offspring. The mesodermal layer (represented by leukocytes) becomes distinct around day 16. Thus, variants present in both leukocytes and colon tissue may also be present in germ cells. In cases, in which the *APC* mosaic variant is restricted to the colon tissue and not detectable in leukocytes it can be presumed that the variant occurred after mesoderm and endoderm specification and therefore is unlikely to be transmitted to offspring.

Somatic mosaicism cases is mainlyreported in patients with an attenuated phenotype according to age at onset and/or polyp burden (65% based on the review by Jansen et al. [14]), even though the position of the pathogenic variant in *APC* would have been expected to result in classical (typical) FAP. Therefore, the offspring of mosaic patients may develop a more severe phenotype than the affected parent, this is relevant for genetic counseling. Surveillance should be performed according to clinical presentation.

Interestingly, a recurrent somatic *APC* splice variant (NM_000038.6: c.835–8 A > G) was observed in patients with unexplained multiple colorectal adenomas and could be associated with colibactin exposure as an alternative pathway of polyp formation and differential diagnosis to hereditary forms of adenomatous polyposis [17].

Based on the publications to date, it can be concluded that *APC* mosaicism is a relevant, but still underreported cause of adenomatous polyposis, especially in cases with a more attenuated phenotype without a family history of polyposis. For those cases (with at least 20 adenomas), in which no germline variant in one of the known polyposis genes could be identified, it is recommended to include *APC* mosaic analysis in routine diagnostic workflows. It also might be reasonable to carefully exclude *APC* mosaicism prior to admittance of this patient group in studies that aim to identify new causative highly penetrant genes.

## *APC* I1307K variant

The *APC* gene plays a crucial role in colorectal cancer (CRC) susceptibility. Most pathogenic variants in *APC* affect Wnt signaling by promoting β-catenin accumulation, leading to polyposis. However, the *APC* I1307K variant (NM_000038.6: c.3920T > A) functions differently. Although located in the β-catenin binding domain, it doesn’t disrupt Wnt signaling, as the protein structure remains mostly intact. This single-nucleotide change extends an AAATAAAA sequence into eight adenosine nucleotides, making it prone to replication slippage and DNA polymerase errors. This variant increases the risk of somatic truncating variants on this allele and localized genomic instability, subtly elevating cancer risk without significantly increasing the polyp burden [18, 19].

Despite extensive study of the I1307K variant across different populations, disagreements persist in literature. First, the I1307K variant is interpreted differently across laboratories, with classifications ranging from “pathogenic” to “variant of uncertain significance” [20]. It has to be noted that the widely used variant interpretation criteria are developed for Mendelian diseases and not for low/moderate penetrant variants, as also discussed in the *APC*-specific variant interpretation criteria [21, 22]. As such, the ClinGen Low Penetrance/Risk Allele Working Group recommend the term “risk allele” [23].

Another key area of debate is the impact of the carrier’s ancestry on cancer risk. These differences have led to a variability in clinical recommendations for carriers and their families. Table 1 outlines the variant classifications and screening recommendations from three leading organizations. This review aims to summarize the pathogenicity data across different ethnicities and explain the basis for the differing recommendations.

**Table 1:** Summary of clinical recommendations for *APC* I1307K variant carriers

Organization	Variant Classification	Screening Recommendations	Additional Notes
National Comprehensive Cancer Network (NCCN)^**a**^	Significant risk factor for CRC in average-risk Ashkenazi Jews	High-quality colonoscopy every 5 years, starting at age 40 or 10 years prior to CRC diagnosis in a first-degree relative.	Applies to all carriers, regardless of ancestry, due to insufficient evidence to support ancestry-specific risk differences. Acknowledges that some individuals may be unaware of their Ashkenazi heritage.
International Society for Gastrointestinal Hereditary Tumours (InSiGHT) [25]	Pathogenic with low penetrance, specifically in Ashkenazi Jewish populations	Colonoscopy every 5 years, starting at age 45–50 for carriers of Ashkenazi descent. Non-Ashkenazi carriers should follow national CRC screening guidelines.	Differentiates recommendations based on ancestry, with additional surveillance limited to Ashkenazi carriers.
UK Cancer Genetics Group (UKCGG) [26]	Does not recommend considering or reporting the variant in NHS-funded diagnostic *APC* testing	Additional surveillance only in the presence of a strong family history of CRC	

^a^ https://www.nccn.org/professionals/physician_gls/pdf/genetics_ceg.pdf

Before reviewing the clinical implications of the I1307K variant based on different ancestries, we want to shed light on relevant ethnic groups by exploring historical and contemporary factors that are pertinent to genetic discourse and may influence surveillance strategies for carriers and their families.

Jewish history has been marked by numerous events that led to the migration of Jewish populations from Jerusalem and its surrounding areas, resulting in their dispersion across various regions all over the world. This resulted in distinct ethnic groups with shared ancestral origins and unique genetic traits influenced by host populations.

Research into the genetic anthropology of the I1307K variant suggests that the variant arose before the beginning of the Jewish diaspora (586BC) [26]. Thus, the observation that the variant is found in both Ashkenazi and non-Ashkenazi individuals is consistent with this timeline. However, the significantly higher prevalence of this variant in the Ashkenazi population still requires further explanation. European Jewish communities migrated within the continent, particularly during the late Middle Ages, due to religious persecution, and established Ashkenazi communities in Eastern Europe [27–29].

In 1648–1649, these communities faced dramatic population declines due to the Cossack massacres. The small remaining communities expanded rapidly between 1765 and 1900, growing from 560,000 to 5 million. This expansion from a small founder population led to a high prevalence of certain genetic variants, including I1307K, through genetic drift [30].

Extensive research over the past two decades has linked the I1307K variant to increased CRC risk in Ashkenazi Jews (AJ). A meta-analysis by Valle et al., covering 19 studies, found that 11.5% of AJ CRC patients carry the I1307K variant, compared to 7.2% of AJs without CRC, with an odds ratio (OR) of 1.68 (95% CI 1.50–1.87; *p* < 0.00001) [24]. However, carriers do not show a higher propensity for multiple adenomas, and the age at first adenoma diagnosis is consistent across both groups [31–33]. One study noted that, after age adjustment, I1307K carriers are more likely to have colorectal polyps than non-carriers (51.3% vs. 33.6%; *p* = 0.03), but the number of polyps did not significantly differ [34].

In contrast to AJ, the definition of Sephardi Jews (SJ) is not based on ethnicity, but on cultural and religious background. SJ, used to live in Spain and Portugal until their expulsion in the late 15th century to North Africa, Western Asia, Southern Europe, and parts of America. Today, many reside in Israel [35]. Data on SJ alone is limited, with most studies grouping all non-AJ together, composed mainly of SJ and Yemenite Jews. Valle et al., based on five case-control studies and three without a control group, found the prevalence of I1307K to be 2.5% (16/651) in non-AJ Jewish CRC patients and 1.8% (20/1097) in controls, showing no significant CRC risk association (OR 1.36, 95% CI 0.65–2.78, *p* = 0.39) [24].

The data summarized above primarily links the increased CRC risk associated with the I1307K variant to AJ populations, aligning with the InSiGHT recommendation for CRC screening focused on AJ carriers [24]. However, we propose several points for consideration in this context, which may support the approach suggesting high-quality colonoscopy every five years for all Jewish carriers.

(1) Studies on SJ have low participant numbers, only 10% of those in AJ I1307K studies, necessitating more data to confirm non-significant results.
(2) Post-Holocaust demographic shifts led to increased migration and intermarriage among Jewish communities. In Israel, intermarriage rates between AJ and non-AJ increased from 9% in the 1950s to 25% and continue to rise, blurring distinctions between populations [36].

Only few studies report the association between CRC and I1307K in non-Jewish populations. In four small cohort studies from European countries (Croatia, Sweden, and England), only one CRC participant carried the variant [37–40], and no CRC was found in another cohort (including Italian, Finnish, and Hawaiian-Japanese individuals) [41]. Conflicting results were seen in population-based case-control studies. Based on Spanish exome array data, Valle et al. cited in their review that the I1307K variant was found to be 0.17% in CRC patients and 0.15% in controls (OR = 1.18, 95% CI 0.34–4.03, p = NS) [24]. On the other hand, Forkosh et al. found the variant in 0.24% of CRC patients and 0.12% of controls among non-Ashkenazi white (NAW) individuals, leading to an OR of 1.95, (95% CI 1.39–2.73, *p* < 0.01) [42]. Pooling data from studies in NAW shows a higher prevalence in CRC patients (0.23%) than controls (0.12%), with an OR of 1.88. However, the results are mainly based on Forkosh’s study and may not apply to all NAW. Three studies assessed the prevalence of I1307K in Israeli Arabs and Egyptians [34, 43, 44], with reported prevalence in CRC patients ranging from 3.1% (Israeli Arabs with CRC in north Israel) [34] to 33% (Israeli Arabs younger than 60 years at a single center) [43].

InSiGHT recommends CRC screening only for AJ I1307K carriers due to lack of conclusive evidence in other ethnicities [24]. On the other hand, the NCCN recommends screening regardless of ethnicity, as some individuals may be unaware of their Ashkenazi heritage. Moreover, as shown, there might be an increased CRC risk in other ethnicities. Further studies are needed to make a definite recommendation. Meanwhile, screening regardless of ethnicity should be considered.

Another controversy between the NCCN and InSiGHT concerns the age to begin screening. The NCCN recommends starting colonoscopy at age 40 (or 10 years before CRC diagnosis in a first-degree relative), while InSiGHT suggests starting at 45–50. Since no evidence exists of early-onset CRC among I1307K carriers, beginning screening at the same age of average risk population sounds reasonable [24].

Data on the effect of I1307K on extracolonic cancer risk are limited. Two Israeli studies showed an increased overall extracolonic cancer risk but did not mention ethnicity [45, 46]. Only two studies assessed this risk in AJ [42, 47], with a meta-analysis finding no increased risk (7% both in extracolonic cancer patients and in controls). Most included patients were from one study [42]. Several studies examined the effect of I1307K on specific cancer types among different ethnicities. Valle et al. found no increased risk for breast, prostate, and pancreatic cancer among AJ carriers, though there was a trend towards a higher prevalence of pancreatic cancer (9.2% in cases vs. 7% in controls, OR 1.36, 95% CI 0.97–1.89, *p* = 0.06) [24]. One large population-based study found higher prevalence for renal cancer (both sexes) and melanoma (males only) among AJ carriers compared to healthy individuals (renal cancer: OR 1.64, 95% CI 1.04–2.47; melanoma: OR 2.04, 95% CI 1.24–3.22, *p* < 0.05) [42]. Data on non-Jewish populations are scarce, with two small studies from Turkey [48, 49] and one large study from the US and Europe [42] indicating higher I1307K rates in melanoma patients (OR 2.54, 95% CI 1.57–3.98, *p* < 0.01), as well as in breast (female only) and prostate (male only) cancer patients (OR 1.73, 95% CI 1.18–2.65, *p* < 0.01; OR 2.42, 95% CI 1.45–3.94, *p* < 0.01, respectively). All three organizations (Table 1) agree that data on extracolonic cancer are controversial and scarce, so currently screening is not recommended.

In summary, strong evidence links the I1307K variant to increased CRC risk in AJ. More data is needed to assess its pathogenicity in non-AJ populations(Jewish and non-Jewish) and its impact on cancer onset and prognosis across different ethnicities. Resolving these issues will clarify screening recommendations for non-AJ populations and the appropriate age to begin screening.

## Gastric adenocarcinoma and proximal polyposis of the stomach (GAPPS)

GAPPS was first described in 2012 [50] and therefore is a relatively new entity. There remains uncertainty regarding many aspects of the condition and its management. It is characterized by the development of fundic gland polyps and an increased risk of gastric cancer. The following clinical diagnostic criteria have been proposed [50], all of which should be fulfilled:

1) gastric polyps restricted to the body and fundus with no evidence of colorectal or duodenal polyposis.

2) > 100 polyps in the proximal stomach in the index case or > 30 polyps in a first-degree relative of another case.

3) predominantly fundic gland polyps (FGPs), some having regions of dysplasia (or a family member with either dysplastic FGPs or gastric adenocarcinoma).

4) an autosomal dominant pattern of inheritance.

Exclusions include other gastric polyposis syndromes and the use of proton pump inhibitors.

Since the original clinical description, three different heterozygous single nucleotide variants in the promoter 1B region of *APC* [NM_001127511: c.-195 A > C (in some cases together with c.-125delA), c.-192 A > G and c.-191T > C] has been discovered as the underlying genetic cause of GAPPS, showing perfect segregation with GAPPS in all six investigated families, including one family with 27 affected individuals. *APC* functional analyses show that these variants interrupt the Ying Yang 1 (YY1) binding site, reducing the expression from promoter 1B [51].

Notably, gastric polyposis and cancer occur in some individuals with large deletions around the promoter 1B. This raises the question of whether this region specifically predisposes individuals to increased gastric cancer risk and a more severe gastric phenotype.; however carpeting gastric polyposis and gastric cancer can be seen in patients with FAP and an underlying pathogenic variant not linked to the promoter 1B region of *APC*. Certainly, large deletions in promoter 1B often have colonic polyposis and have a very variable phenotype. So, the large bowel and gastric findings overlap with FAP, and it is not clear if the gastric polyposis or cancer risk in those with a promoter 1B deletion differs to FAP due to other pathogenic variants. Indeed, it isn’t clear whether there is a difference in the mechanisms and natural history of gastric polyposis and gastric cancer in GAPPS compared to FAP.

There are data to understand why single nucleotide variants in *APC* promoter 1B cause an isolated (or at least predominant) gastric phenotype. Transcription from the promoter 1B is much higher than from the promoter 1 A in gastric mucosa, which might explain why GAPPS-affected families carry promoter 1B variants and the absence of a colonic phenotype is explained by the colonic cells being protected by the expression of the 1 A isoform [51–53].

### As mentioned earlier, there are remaining areas of uncertainty

#### Penetrance

The penetrance of gastric cancer in GAPPS is not well established. Given the rarity of the condition, it is likely that the elevated risk described in the literature is subject to ascertainment bias. A recent review has been performed including a total of 113 patients from 27 different families, all of them (obligate) carriers of pathogenic variants in the *APC* promotor 1B; in addition to the variants mentioned above the variant c.-191T > G is reported [54]. The mean age at diagnosis was 43 years (range 10 to 92 years). Fundic gland polyposis was not observed in 9.3%. Data regarding gastrectomy were available in 81, of whom 37 (46%) underwent gastrectomy (either for cancer or prophylactically). Gastric cancer data were available for 103; 31/103 patients (30%) had gastric cancer at a median age of 45 years (range 19–75). So, the penetrance is not complete but beyond that determining how high the penetrance truly is, remains very difficult.

#### Extra-gastric phenotype

It was originally proposed that there should be no duodenal or large bowel polyposis as part of the clinical diagnostic criteria. Exactly what constitutes polyposis is not clarified and can be debated with the increasing understanding of the breadth of severity of polyp burden in the large bowel of those with FAP. For the duodenum it is even more difficult. The Spigelman classification system is used but what stage of polyposis would count as duodenal polyposis? A solitary duodenal adenoma gives stage I disease - is this sufficient to count as duodenal polyposis? Also, it is clear that the risk of developing duodenal adenomas in FAP increases with age, yet there is no defined age at which an absence of duodenal adenomas should be assessed to prove exclusion.

Unfortunately, much of the published data do not give a full description of large bowel or duodenal phenotype and these data have not been systematically collected. In one systematic review [54], 19/46 (41%) patients had colorectal polyps. All presented with less than 20 either hyperplastic or adenomatous polyps. So, there may be a large bowel phenotype, although a low polyp burden which may overlap with FAP and indeed sporadic colonic polyps. One patient developed colorectal cancer, although this was at age 75 years. No duodenal polyposis has been described in the limited patients where duodenal phenotype is described. Of course, in those undergoing gastrectomy, it is not possible to perform duodenal surveillance with standard endoscopy, unless specific surgical modifications are undertaken to allow endoscopic access to the duodenum. So, it is impossible to know if any of these patients did subsequently develop duodenal adenomas, although there are no reports of the development of duodenal cancer.

#### Management

There are no established clinical guidelines for GAPPS. If gastric cancer is diagnosed, then this should be managed according to standard practice. Other than this aspect, there is little clarity about the clinical management of GAPPS.

It is widely agreed that upper GI endoscopy surveillance is indicated but when to start and how often it should be performed are not established. Developing meaningful guidelines is hampered by the lack of understanding as to the natural history of the condition. Endoscopic polypectomy may be performed in an attempt to either manage the condition and/or to detect dysplasia earlier. Good prospective data on the outcome of surveillance are lacking and interpreting historical data where surveillance is not performed in a standardised manner is difficult.

There is significant variation in disease expression even within families. As such, a personalised approach would seem to be appropriate. However, there are no prospectively collected surveillance data to help inform how to personalise gastric surveillance.

Similarly, gastrectomy will need to be considered in some patients but the indications for this are not clear. The finding of any dysplasia in the stomach, irrespective of the overall burden of polyposis? Or a subjective assessment that the stomach is not amenable to meaningful surveillance, even in the absence of dysplasia? Timing of such surgery is difficult, and the sequelae of gastrectomy make this decision making more complex. That said, cancer prevention should be the primary goal, as early detection may be extremely difficult/impossible in the setting of florid gastric polyposis. If one looks at gastric cancer in the setting of FAP, where gastric cancer arose in a similar setting of proximal florid polyposis, the outcomes have been reported to be extremely poor [55].

Should large bowel surveillance be offered? Finding colonic polyps appears to be far more frequent than the original clinical description of the condition would have one believe. It is not clear however, as to the relative risk compared to the general population. It would seem prudent to perform colonoscopy but whether this should be a one-off evaluation or repeated (e.g. 5 yearly as one would for at risk relatives of someone with a genetically undefined but clinical diagnosis of adenomatous polyposis) if the baseline is normal remains open to debate.

In summary, GAPPS remains a clinical challenge. Our understanding of the condition, its natural history remains suboptimal. This along with the wide phenotypic variability means that developing practice guidelines is challenging. Given the rarity of GAPPS, international collaboration to collect prospective surveillance data will be informative. Particularly focusing on those who are identified through cascade testing, will give a better understanding and less bias assessment of disease penetrance.

## Data Availability

No datasets were generated or analysed during the current study.
